# Association between electromagnetic field exposure and abortion in pregnant women living in Tehran

**Published:** 2017-02

**Authors:** Seyed Mohammad Javad Mortazavi, Seyed Alireza Mortazavi, Maryam Paknahad

**Affiliations:** 1 *Medical Physics Department, School of Medicine, Shiraz University of Medical Sciences, Shiraz, Iran*; 2 *Ionizing and Non-ionizing Radiation Protection Research Center (INIRPRC), Shiraz University of Medical Sciences, Shiraz, Iran*; 3 *Student of Research Committee, School of Medicine, Shiraz University of Medical Sciences, Shiraz, Iran*; 4 *Dentomaxillofacial* * Radiology Department,* * School of Dentistry, Shiraz University of Medical Sciences, Shiraz, Iran*

**Dear Editor**

With great interest, we have read the article by Abad *et al.* entitled “Association between electromagnetic field exposure and abortion in pregnant women living in Tehran” that is published in International Journal of Reproductive BioMedicine Vol. 14. No. 5. pp: 347-354, May 2016. In this article, the authors evaluated the possible associations between electromagnetic waves exposure level and the rate of miscarriage in pregnant women. The electromagnetic radiation, in this study, had a significant association with the increased abortion in women who were exposed to these radiations. These findings were based on the measurements of electromagnetic waves within the residential locations of the 413 samples, very close to the entrance door of their home, according to the standard instructions of ICNIRP. Over the past several years, our laboratories at the Ionizing and Non-ionizing Radiation Protection Research Center (INIRPRC) have expanded their focus on studying the health effects of exposure to some common and/or occupational sources of electromagnetic fields (EMFs) such as cellular phones (1-9), mobile base stations (10), mobile phone jammers (11, 12), laptop computers (13), radars (2), dentistry cavitrons (14) and MRI (15, 16). Although the paper authored by Abad *et al.* is a well-structured article and addresses a very challenging issue, it has some major shortcomings. The first shortcoming of this paper comes from this cardinal point that the authors have simply ignored the role of exposure to extremely low frequency EMFs (e.g. exposure of the pregnant women living in houses close to power lines). It is worth noting that the NARDA SRM-3000 used in their study operates in the frequency range of 27MHz-3GHz and cannot measure extremely low frequency EMFs. It is also worth mentioning that previous studies conducted in Iran indicated that the exposure to extremely low frequency electromagnetic fields is probably related to early spontaneous abortions (17). Another shortcoming of this paper comes from ignoring the role of mobile phone/cordless phone use by pregnant women in evaluation of the risk of abortion. It should be noted that some studies conducted in Iran showed that the use of mobile phones can be linked to the early spontaneous abortions (18). We hope that our comments help better understanding of the effects of EMF on the pregnancy outcome.
