# Supplementary material for: Association between electromagnetic field exposure and abortion in pregnant women living in Tehran
Source: Int J Reprod Biomed. 2017 Feb;15(2):115–6. (PMC5405225)
Supplement: Related texts

# Author reply

**Published:** 2017-02

**Dear Editor**

We are greatly thankful for the precious comments of the esteemed commentator, Dr. Mortazavi. Hereby, you can find my peer-to-peer response to his worthy comments: 

First comment: As it is clear, our research is focuses on exposed electromagnetic waves rather than fields. We know that in the case of ELF, according to low frequency of 50 Hz, any wave radiation is impossible from low length cables or other electromagnetic sources, and only electric and magnetic inductions effect the receptor, that are all totally out of the scope of this article. Hence, the ELF frequency range is not included in the study with intent, so the comment should crucially not be considered as shortcoming of the study. 

Second comment: It is obvious that the article focuses on downlink electromagnetic waves which are special types of electric field coupled with magnetic field radiating in the speed of light, which means electromagnetic waves down-coming from electromagnetic sources as the panel antennas installed on the cellphone towers, television broadcast towers, etc. to the human as subjects of susceptibility. In all measurements shown in the article, the downlink frequency range is set in Narda measuring instrument, not the uplink frequency ranges which are radiated from mobile phones or the other radiating devices used as accessories or laptop and so on mentioned at commentator's reference 18. As you know, so called uplink frequencies are the same for all samples studied, so can be rightly excluded from the study. Therefore the uplink frequency range is not set with intent. 

## References

[1] Mortazavi SMJ, Motamedifar M, Namdari G, Taheri M, Mortazavi AR, Shokrpour N (2014). Non-Linear Adaptive Phenomena which Decrease the Risk of infection after Pre-Exposure to Radiofrequency Radiation. Dose-Response.

[2] Mortazavi SMJ, Taeb S, Dehghan N (2013). Alterations of Visual Reaction Time and Short Term Memory in Military Radar Personnel. Iran J Publ Health.

[3] Mortazavi SMJ, Rouintan MS, Taeb S, Dehghan N, Ghaffarpanah AA, Sadeghi Z (2012). Human short-term exposure to electromagnetic fields emitted by mobile phones decreases computer-assisted visual reaction time. Acta Neurologica Belgica.

[4] Mortazavi SMJ, Mosleh-Shirazi MA, Tavassoli AR, Taheri M, Mehdizadeh AR, Namazi SAS (2013). Increased Radioresistance to Lethal Doses of Gamma Rays in Mice and Rats after Exposure to Microwave Radiation Emitted by a GSM Mobile Phone Simulator. Dose-Response.

[5] Mortazavi S, Mosleh-Shirazi M, Tavassoli A, Taheri M, Bagheri Z, Ghalandari R (2011). A comparative study on the increased radioresistance to lethal doses of gamma rays after exposure to microwave radiation and oral intake of flaxseed oil. Iran J Radiat Res.

[6] Mortazavi SMJ, Habib A, Ganj-Karimi AH, Samimi-Doost R, Pour-Abedi A, Babaie A (2009). Alterations in TSH and Thyroid Hormones Following Mobile Phone Use. OMJ.

[7] Mortazavi SMJ, Daiee E, Yazdi A, Khiabani K, Kavousi A, Vazirinejad R (2008). Mercury release from dental amalgam restorations after magnetic resonance imaging and following mobile phone use. Pak J Biol Sci.

[8] Mortazavi SMJ, Ahmadi J, Shariati M (2007). Prevalence of subjective poor health symptoms associated with exposure to electromagnetic fields among University students. Bioelectromagnetics.

[9] Mortazavi SMJ, Motamedifar M, Namdari G, Taheri M, Mortazavi AR (2013). Counterbalancing immunosuppression-induced infections during long-term stay of humans in space. J Med Hypotheses Ideas.

[10] Mortazavi SMJ (2013). Safety issue of mobile phone base stations. J Biomed Physic Eng.

[11] Mortazavi S, Parsanezhad M, Kazempour M, Ghahramani P, Mortazavi A, Davari M (2013). Male reproductive health under threat: Short term exposure to radiofrequency radiations emitted by common mobile jammers. J Hum Reprod Sci.

[12] Parsanezhad ME, Mortazavi SMJ, Doohandeh T (2015). Exposure to Radiofrequency Radiation Emitted from Mobile Phone Jammers Adversely Affects the Quality of Human Sperm. Int J Radiat Res.

[13] Mortazavi SMJ, Tavasoli AR, Ranjbari F, Moamaei P (2011). Effects of Laptop Computers' Electromagnetic Field on Sperm Quality. J Reprod Infertil.

[14] Mortazavi SM, Vazife-Doost S, Yaghooti M, Mehdizadeh S, Rajaie-Far A (2012). Occupational exposure of dentists to electromagnetic fields produced by magnetostrictive cavitrons alters the serum cortisol level. J Nat Sci Biol Med.

[15] Mortazavi SM, Paknahad M (2016). Effect of magnetic resonance imaging on microleakage of amalgam restorations: an in vitro study. Dentomaxillofac Radiol.

[16] Mortazavi SMJ, Neghab M, Anooshe SMH, Bahaeddini N, Mortazavi G, Neghab P (2014). High-field MRI and Mercury release from dental amalgam fillings. THE IJOEM.

[17] 1Shamsi Mahmoudabadi F, Ziaei S, Firoozabadi M, Kazemnejad A (2013). Exposure to extremely low frequency electromagnetic fields during pregnancy and the risk of spontaneous abortion: a case-control study. J Res Health Sci.

[18] Mahmoudabadi FS, Ziaei S, Firoozabadi M, Kazemnejad A (2015). Use of mobile phone during pregnancy and the risk of spontaneous abortion. J Environment Health Sci Eng.
